# Age-dependent differences in SARS-CoV-2 entry-factor expression and innate immune responses between pediatric and adult liver organoids

**DOI:** 10.3389/fpubh.2026.1951670

**Published:** 2026-10-23

**Authors:** Tianchen Xue, Jie Hu, Zhe Liu, Lefan Zhu, Yi Zhao, Xiaoxue Ren, Lixia Xu, Xiaoxing Li, Jing Lu, Lina Wang

**Affiliations:** 1 Department of Pathology, The First Affiliated Hospital, Sun Yat-sen University, Guangzhou, China; 2 Department of Oncology, The First Affiliated Hospital, Sun Yat-sen University, Guangzhou, Guangdong, China; 3 Guangdong Provincial Center for Disease Control and Prevention, Guangzhou, China; 4 Institute of Precision Medicine, The First Affiliated Hospital, Sun Yat-sen University, Guangzhou, China

**Keywords:** age-dependent host responses, innate immune response, pediatric liver organoids, SARS-CoV-2, viral entry factors

## Abstract

**Background:**

Since early 2022, a global surge in cases of severe acute hepatitis of unknown origin (AHUO) in children has been reported, with no definitive aetiology established. Epidemiological evidence suggests a potential link to SARS-CoV-2 infection, particularly during Omicron-predominant periods, yet the mechanisms underlying the apparently higher susceptibility of children to SARS-CoV-2-related liver injury remain poorly understood. This study used non-tumorous (tumor-adjacent) liver organoids derived from pediatric and adult donors to compare viral RNA accumulation and host immune responses to SARS-CoV-2 wild-type (WT) and Omicron (BA.1/BA.2) variants, aiming to explore the possible role of SARS-CoV-2 in pediatric acute hepatitis.

**Methods:**

Liver organoids were established from non-tumorous (tumor-adjacent) liver tissue of three adult patients with hepatocellular carcinoma and three pediatric patients with hepatoblastoma, collected between February and December 2022. Organoids were infected with SARS-CoV-2 WT, Omicron BA.1 or BA.2 at a multiplicity of infection (MOI) of 0.1. Viral loads in supernatants and intracellular viral RNA were quantified by RT-qPCR at 24 and 48 h post-infection (hpi). At 48 hpi, RNA-seq was performed to profile transcriptomic changes and pathway enrichment. Additionally, single-cell RNA-seq data were used to compare baseline expression of SARS-CoV-2 entry-associated genes between pediatric and adult hepatocytes.

**Results:**

Pediatric hepatocytes showed significantly higher basal expression of entry-related genes, including ASGR1, KREMEN1, CTSB, CTSL, and VIM, than adult cells. Both pediatric and adult liver organoids were permissive to WT and Omicron, but Omicron BA.1/BA.2 yielded higher intracellular viral RNA levels than WT, with notably higher intracellular viral RNA burden in pediatric organoids. RNA-seq revealed that Omicron infection induced a more pronounced innate-immune-associated transcriptional response in pediatric organoids—encompassing interferon signaling, RIG-I-like receptor and Toll-like receptor pathways—alongside relative downregulation of adaptive immune-pathway transcripts. In adult organoids, transcriptomic divergence between Omicron and WT was less pronounced. Compared with adults, Omicron-infected pediatric organoids upregulated interferon-γ signaling and other innate immune-associated transcriptional programs, while downregulating adaptive immune-pathway transcripts; key receptors such as ACE2 and TMPRSS4 remained consistently higher in pediatric organoids across all viral strains.

**Conclusion:**

Pediatric liver organoids accumulate higher levels of Omicron viral RNA than WT-infected organoids and mount a stronger innate-immune transcriptional response alongside relative downregulation of adaptive immune-pathway transcripts, correlating with elevated expression of viral entry factors. These age-dependent *in vitro* differences raise the hypothesis that SARS-CoV-2, particularly Omicron, could contribute to hepatocyte injury in children; however, this organoid-based model does not include patients with acute hepatitis of unknown origin and cannot establish a causal or epidemiological link, which will require dedicated prospective clinical and mechanistic studies.

## 1 Introduction

Since the initial report of acute hepatitis of unknown origin (AHUO) in children from the United Kingdom in March 2022, more than 1,010 probable cases have been identified across 35 countries, representing a significant global pediatric health concern (1, 2). The World Health Organization (WHO) defines a probable case as an individual aged 16 years or younger presenting with acute hepatitis (serum alanine aminotransferase (ALT) or aspartate aminotransferase (AST) > 500 U/L) not caused by hepatitis viruses A–E, with common causes such as medications or toxins ruled out (3). Clinically, the disease spectrum ranges from gastrointestinal symptoms and jaundice to acute liver failure (ALF), necessitating liver transplantation in severe instances and resulting in fatal outcomes in some cases (2, 3). The absence of a confirmed causative agent underscores the critical need to elucidate its pathogenesis.

Early epidemiological investigations consistently excluded conventional hepatitis viruses. A high detection rate of human adenovirus (HAdV), particularly in pediatric patients with severe hepatitis, has been documented, though a definitive causal link remains unestablished (3). Concurrently, the COVID-19 pandemic revealed that SARS-CoV-2 infection can induce multi-organ dysfunction, including hepatic injury, primarily observed in adults (4, 5). Proposed mechanisms of SARS-CoV-2-related liver damage include direct viral cytopathy, immune-mediated injury, drug-induced toxicity and ischaemia (4). These observations led to the hypothesis that AHUO may be linked to prior infection with pathogens such as HAdV or SARS-CoV-2, potentially triggering an aberrant immune response, especially in children with altered immune exposure following pandemic restrictions (6).

Emerging evidence supports a potential association with SARS-CoV-2. A reported increase in non-A–E hepatitis incidence since the pandemic onset is consistent with this possibility (7). Case studies have documented pediatric severe hepatitis following COVID-19, and cohort analyses of AHUO patients show a notable prevalence of concurrent or prior SARS-CoV-2 infection, as indicated by PCR positivity (8.9–12.8%) or nucleocapsid IgG seropositivity (up to 52%) (3). Furthermore, detection of SARS-CoV-2 antigens within liver tissue, particularly in areas of immune infiltration, suggests that COVID-19 could drive AHUO, potentially as a form of post-acute sequelae of SARS-CoV-2 infection (PASC) affecting the liver (8). Epidemiological observations also indicate a temporal correlation between community surges of the SARS-CoV-2 Omicron variant (B.1.1.529) and clusters of AHUO cases in children (9). Despite these associations, the precise role of SARS-CoV-2 in the aetiology of pediatric hepatitis, and the mechanistic basis for its apparently heightened severity in children compared with adults, remain unclear.

To address these gaps, we investigated the potential link between SARS-CoV-2 and AHUO by comparing viral RNA dynamics in pediatric and adult liver models. Using organoids derived from non-tumorous (tumor-adjacent) liver tissue, we assessed the viral RNA kinetics of SARS-CoV-2 WT and Omicron (BA.1, BA.2) variants. Our findings demonstrate a significantly higher intracellular viral RNA burden of Omicron variants in pediatric liver organoids than in adult models, a distinction not observed with the WT strain. Complementary transcriptional profiling of liver samples from pediatric and adult patients revealed distinct immunopathological landscapes, enabling in-depth characterization of the hepatic immunological landscape. This work provides novel experimental insight into age-dependent features that may contribute to higher susceptibility of children to SARS-CoV-2-associated liver pathology and delineates the underlyin g host-response differences.

## 2 Materials and methods

### 2.1 Patient inclusion and ethics

This study was approved by the Ethics Committee of the First Affiliated Hospital of Sun Yat-sen University, and written informed consent was obtained from participants or their legal guardians. Adult liver organoids were derived from non-tumorous (tumor-adjacent) liver tissue of patients with primary liver cancer who underwent surgical resection. Pediatric liver organoids were derived from non-tumorous (tumor-adjacent) liver tissue of patients with hepatoblastoma. Detailed donor demographic, clinical and pathological characteristics, including viral hepatitis serology, liver fibrosis stage, steatosis, and prior systemic therapy, are summarized in Supplementary Table S1.

### 2.2 Viruses and biosafety

The SARS-CoV-2 viruses included the WT strain (isolate Guangdong/20SF014/2020; EPI_ISL_403934) and the Omicron subvariants BA.1 and BA.2. All strains were obtained from the Guangdong Provincial Center for Disease Control and Prevention. Viruses were propagated and handled exclusively within a Biosafety Level 3 (BSL-3) facility.

### 2.3 Organoid culture

Adult or pediatric liver tissues were dissected into approximately 1 cm^3^ pieces and chopped into small fragments. Fragments were placed in Advanced DMEM/F12 (GIBCO) containing collagenase type IV (GIBCO) and the metalloprotease dispase (Corning) and incubated at 37 °C for 1 h with gentle shaking to dissociate into single cells. After filtration through a 70-μm cell strainer, hepatocytes were resuspended in 200 μL of growth factor-reduced Matrigel (Corning) and plated as several 30-μL droplets in a 48-well tissue-culture plate. Organoids were cultured in expansion medium for 5 days, then switched to differentiation medium for a further 15 days to differentiate into hepatocytes, with medium changed every 5 days. Expansion medium comprised Advanced DMEM/F12 supplemented with 1% penicillin/streptomycin, 1% GlutaMAX, 10 mM HEPES, 1:50 B27 (without vitamin A), 1:100 N2, 1.25 mM N-acetyl-L-cysteine, 10% (vol/vol) Rspo-1-conditioned medium, 10% (vol/vol) WNT3a-conditioned medium, 10 mM nicotinamide, 10 nM gastrin I, 50 ng/mL recombinant human EGF, 100 ng/mL recombinant human FGF10, 25 ng/mL recombinant human HGF, 10 μM forskolin, 5 μM A83-01, 25 ng/mL Noggin and 10 μM Y-27632. Differentiation medium was the expansion medium supplemented with 10 μM DAPT, 3 μM dexamethasone, 25 ng/mL BMP7 and 100 ng/mL recombinant human FGF19.

### 2.4 SARS-CoV-2 infection of organoids

Organoids were infected at a multiplicity of infection (MOI) of 0.1 in organoid culture medium. After 1 h of virus–organoid co-incubation (adsorption), medium was removed and organoids were washed twice with ice-cold PBS to remove unattached virus, then re-embedded in Matrigel and maintained in culture medium.

### 2.5 Real-time PCR analysis of SARS-CoV-2

Viral RNA was extracted from both supernatant and organoid samples using the Viral RNA Mini Kit (Qiagen, Germany). Relative viral loads were determined with a SARS-CoV-2 quantitative reverse-transcription PCR (RT-qPCR) kit (Daan Gene, Guangzhou, China) and analyzed by the ΔΔCT method.

### 2.6 Single-cell RNA sequencing

The scRNA-seq data re-analyzed in this study were retrieved from published work, corresponding to normal peritumor liver tissues from 6 hepatoblastoma (HB) patients (Wang et al., 2023, Cell Reports Medicine 4) and 9 hepatocellular carcinoma (HCC) patients. The procedure of scRNA-seq raw data processing and unsupervised clustering was performed as previously reported (ref: Wang et al., Intratumoral erythroblastic islands restrain anti-tumor immunity in hepatoblastoma. 2023, Cell Reports Medicine 4, May 16, 2023). Raw sequencing reads were aligned to the GRCh38 human reference genome using CellRanger (version 5.0.1) to generate a raw gene expression matrix for each sample. Cells that met the following criterion were regarded as low-quality cells and were removed: (1) total expressed genes <200, (2) proportions of expressed mitochondrial genes of more than 25%. DoubletFinder was applied to each sequencing library to remove potential doublets. Unsupervised clustering was based on the normalized and scaled data using the Seurat R package (version 4.1.0). For non-malignant cell sub-clustering, we performed batch effect correction by the Harmony package (version 0.1.0) of R based on the top 30 PCA components identified. Cell clusters highly expressing hepatocytes marker genes, such as ALB, APOC1, et al., were selected for further analysis. To avoid pseudoreplication caused by treating individual cells as independent observations, we aggregated raw UMI counts at the donor level to perform pseudobulk analysis. Differential expression was tested using DESeq2/edgeR on the pseudobulk count matrix.

### 2.7 RNA-seq analysis

Total RNA was extracted from organoids with the RNeasy Mini Kit (Qiagen, Germany). Oligo(dT) beads (Invitrogen) were used to isolate poly(A) mRNA. Fragmentation buffer was added to truncate mRNA into short fragments, which served as templates for first-strand cDNA synthesis with random hexamer primers. Second-strand cDNA was synthesized using buffer, dNTPs, RNase H and DNA polymerase I. Short fragments were purified with the QIAquick PCR Extraction Kit (Qiagen), redissolved in EB buffer for end repair and poly(A) addition, and ligated to sequencing adaptors. Suitable fragments selected by agarose gel electrophoresis were used as templates for PCR amplification. Libraries were sequenced on the HiSeq X Ten platform, generating 150-bp paired-end reads.

### 2.8 Statistics

Error bars indicate S. D. (for RT-qPCR). For all assays, n refers to the number of independent, donor-derived biological replicates (three pediatric and three adult organoid lines), each represented by the mean of technical triplicates unless stated otherwise in the figure legend. Normality was assessed with the Shapiro–Wilk test and variance homogeneity with Levene’s test; normally distributed data were compared using two-tailed unpaired Student’s *t*-test (two groups) or one-way/two-way ANOVA with Tukey’s *post hoc* test (≥ 3 groups), while non-normally distributed data were analyzed with the Mann–Whitney *U* or Kruskal–Wallis test with Dunn’s correction. For comparisons involving more than two genes or conditions simultaneously (e.g., Figure 1), *p*-values were adjusted for multiple comparisons using the Benjamini–Hochberg false-discovery-rate procedure. Differential expression between pediatric and adult donors in single-cell RNA-seq data was additionally assessed using a donor-level pseudobulk approach (aggregating counts per donor and testing with DESeq2/edgeR) to avoid treating individual cells as independent biological replicates.

**Figure 1:**
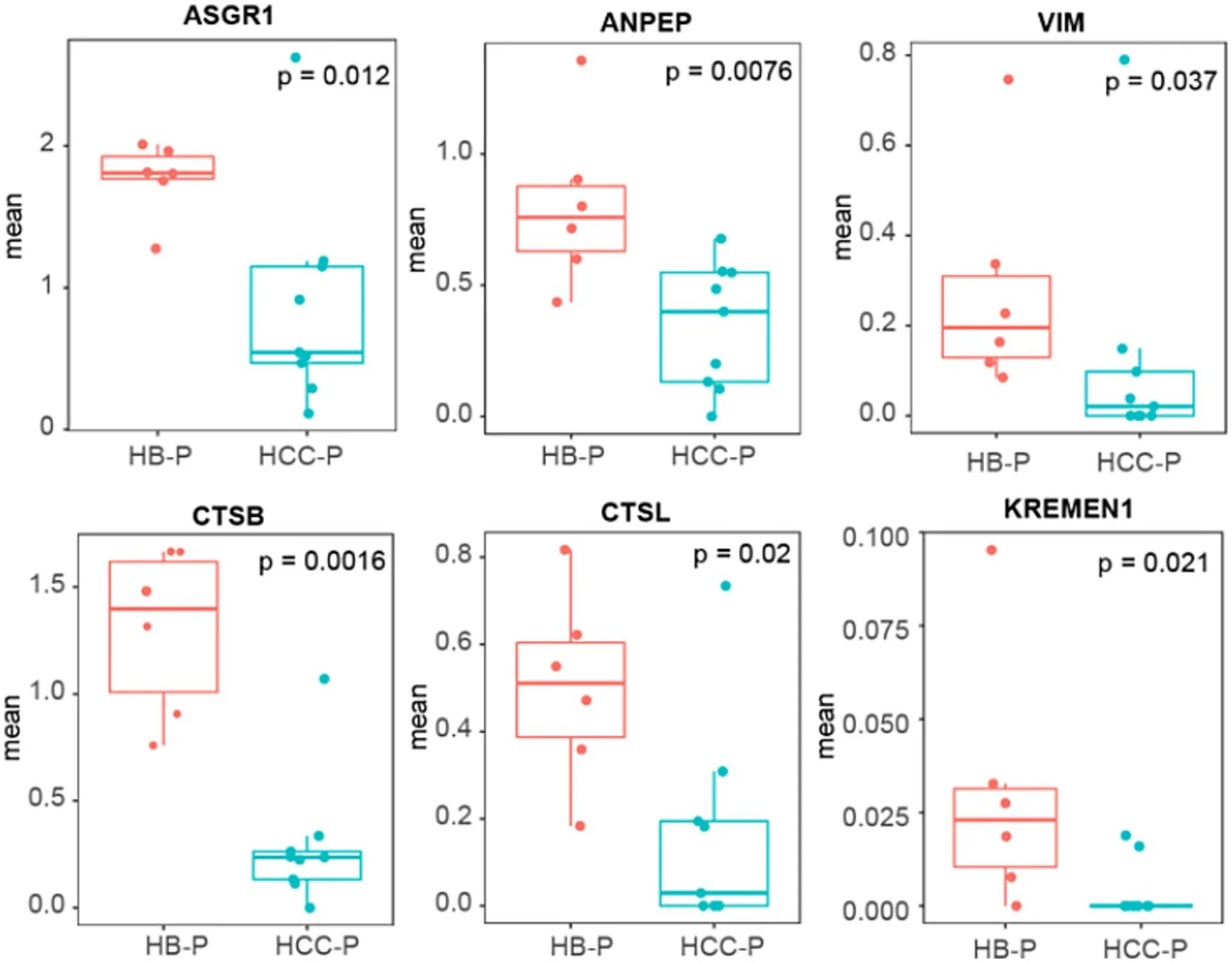
Differential expression of SARS-CoV-2 entry-related genes in hepatocytes from hepatoblastoma (HB) versus hepatocellular carcinoma (HCC) peritumor liver tissues. Boxplots compare expression of ASGR1, ANPEP, VIM, CTSB, CTSL and KREMEN1. Each dot represents an individual patient donor. To avoid pseudoreplication, raw UMI counts of hepatocytes were aggregated per donor for donor-level pseudobulk analysis. Differential expression was assessed using DESeq2. *p*-values were corrected for multiple-gene testing by the Benjamini–Hochberg false-discovery-rate procedure; adjusted *p*-values are shown.

## 3 Results

### 3.1 Differential expression of SARS-CoV-2-related receptors and proteases in pediatric versus adult liver cells

To explore the potential association between SARS-CoV-2 infection and AHUO in children, we compared the expression of SARS-CoV-2-associated host genes using scRNA-seq data from pediatric and adult donors. Several key genes—ASGR1, ANPEP, VIM, CTSB, CTSL and KREMEN1—were expressed at significantly higher levels in pediatric than adult hepatocytes (Figure 1). Notably, ASGR1 and KREMEN1, recently identified as alternative receptors facilitating SARS-CoV-2 entry into liver cells (10–12), were markedly upregulated in children’s liver cells, implying greater intrinsic susceptibility. The proteases CTSB and CTSL, reported as alternative entry pathways whose inhibition produces synergistic antiviral effects (13), were also increased, further supporting enhanced susceptibility of pediatric liver tissue. Extracellular vimentin (VIM), an attachment factor facilitating SARS-CoV-2 entry into human endothelial cells (14, 15), was likewise more highly expressed in children’s liver cells. These findings provide molecular evidence that may explain why children’s livers are more vulnerable to SARS-CoV-2, potentially contributing to AHUO pathogenesis.

### 3.2 Establishment of liver organoids from pediatric and adult donors

To further investigate the age-dependent differences in hepatic susceptibility observed in tissues, we established liver organoids from non-tumorous (tumor-adjacent) liver tissue of pediatric and adult donors as an *in vitro* model, complementing and extending the scRNA-seq findings. We previously showed that SARS-CoV-2 can efficiently infect and replicate in human liver organoids (16). Here we generated three adult and three pediatric liver organoid lines, all from non-tumor adjacent liver tissues of adult HCC and pediatric hepatoblastoma patients, respectively. All lines displayed stable morphological features, confirming their suitability for downstream infection assays (Figure 2A).

**Figure 2:**
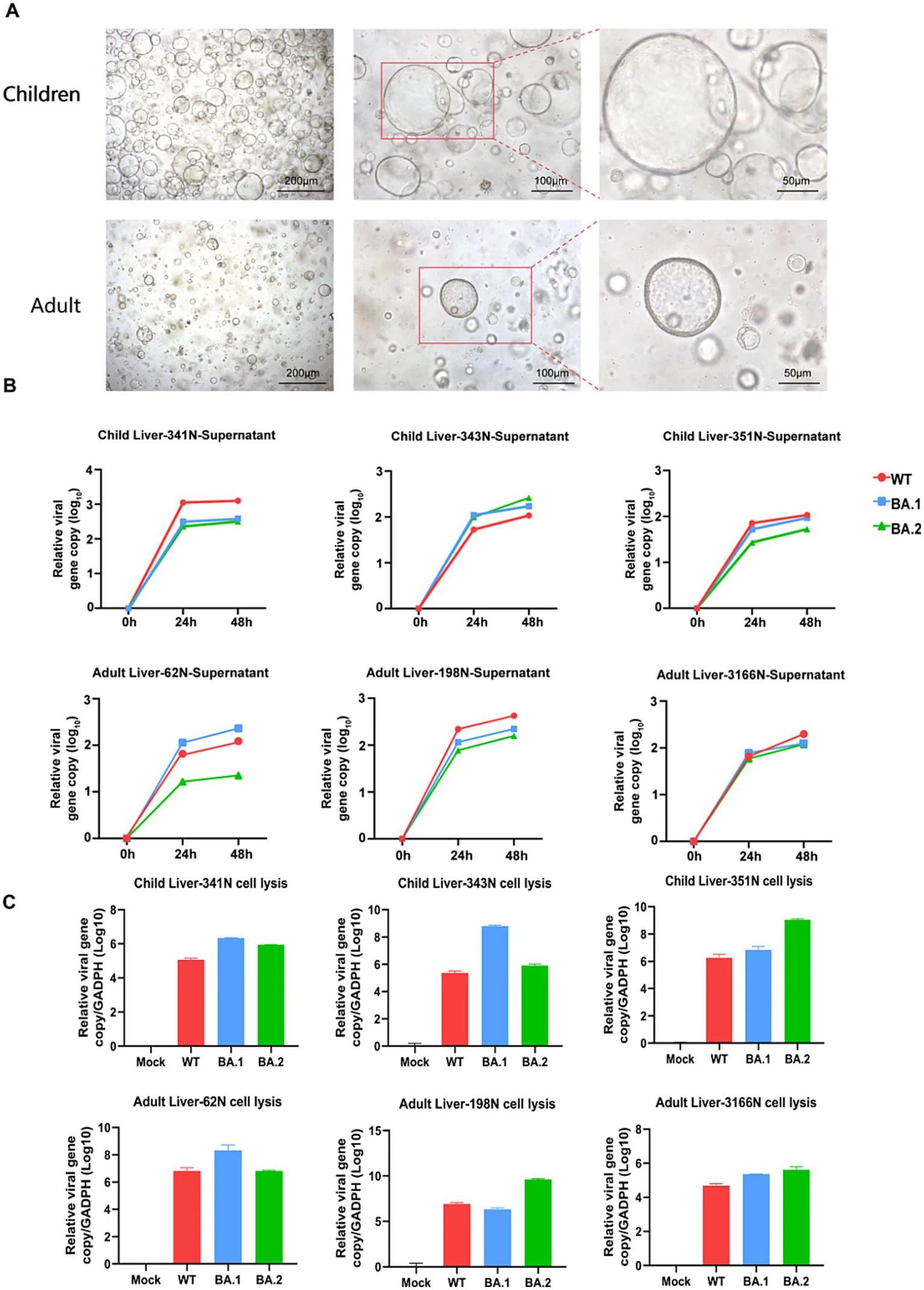
SARS-CoV-2 WT and Omicron infection in pediatric and adult human liver organoids. **(A)** Representative bright-field images of pediatric and adult liver organoids showing typical spherical morphology before infection. **(B)** Viral RNA dynamics in supernatants after infection at MOI 0.1; supernatants collected at 0, 24 and 48 hpi and relative viral RNA abundance quantified by RT-qPCR. **(C)** Intracellular viral RNA in infected organoids (pediatric: Child Liver-341 N, 343 N, 351 N; adult: Adult Liver-62 N, 198 N, 3166 N), lysed at 48 hpi, quantified by RT-qPCR and normalized to GAPDH.

### 3.3 Detection of intrahepatic SARS-CoV-2 infection in liver organoids

To assess susceptibility, liver organoids from SARS-CoV-2-negative donors were exposed to live SARS-CoV-2 WT and Omicron BA.1/BA.2 at an MOI of 0.1, following the 1-h adsorption-and-wash protocol described in Methods. Supernatants were collected at 24 and 48 h post infection (hpi), and organoids were harvested at 48 hpi. Viral load (relative viral RNA abundance) in supernatants increased dramatically by 24 hpi and remained stable at 48 hpi (Figure 2B), demonstrating that human liver organoids are susceptible to SARS-CoV-2 and accumulate substantial viral RNA in supernatants and cell lysates. Intracellular SARS-CoV-2 RNA quantified at 48 hpi showed significant heterogeneity between patient samples. Across three pediatric and three adult lines, both Omicron BA.1 and BA.2 exhibited similar or higher intracellular viral RNA than WT (Figure 2C), indicating a higher intracellular viral RNA burden with Omicron than WT in both pediatric and adult tissues. Together, these findings indicate that both WT and Omicron BA.1/BA.2 infect liver organoids, with Omicron BA.1/BA.2 showing higher intracellular viral RNA levels than WT.

### 3.4 Omicron BA.1/BA.2 induces a stronger immune response than WT in pediatric liver organoids

To uncover mechanisms of Omicron-induced liver injury in children, we performed RNA-seq on control, WT- and Omicron BA.1/BA.2-infected organoids from three children and three adults at 48 hpi. Principal components analysis (PCA) showed clustering of biological replicates within infection groups, and SARS-CoV-2 infection induced a marked transcriptional shift relative to mock controls (Figure 3A, upper). WT and Omicron (BA.1/BA.2) profiles clustered closely, indicating relatively minor differences between variants (Figure 3A, lower). Gene Ontology (GO) and Kyoto Encyclopedia of Genes and Genomes (KEGG) analyses showed that SARS-CoV-2 infection (WT or Omicron) promoted multiple pathways, including GPCR signaling, viral mRNA translation and apoptosis, and notably induced immune pathways such as the adaptive immune system, human T-cell leukemia virus 1 infection, IL-17 signaling, interleukin-1 processing and Toll-like receptor signaling (Figure 3B, upper), while downregulating cell-adhesion molecules (CAMs) and lipid, cholesterol and fatty-acid metabolism (Figure 3B, lower).

**Figure 3:**
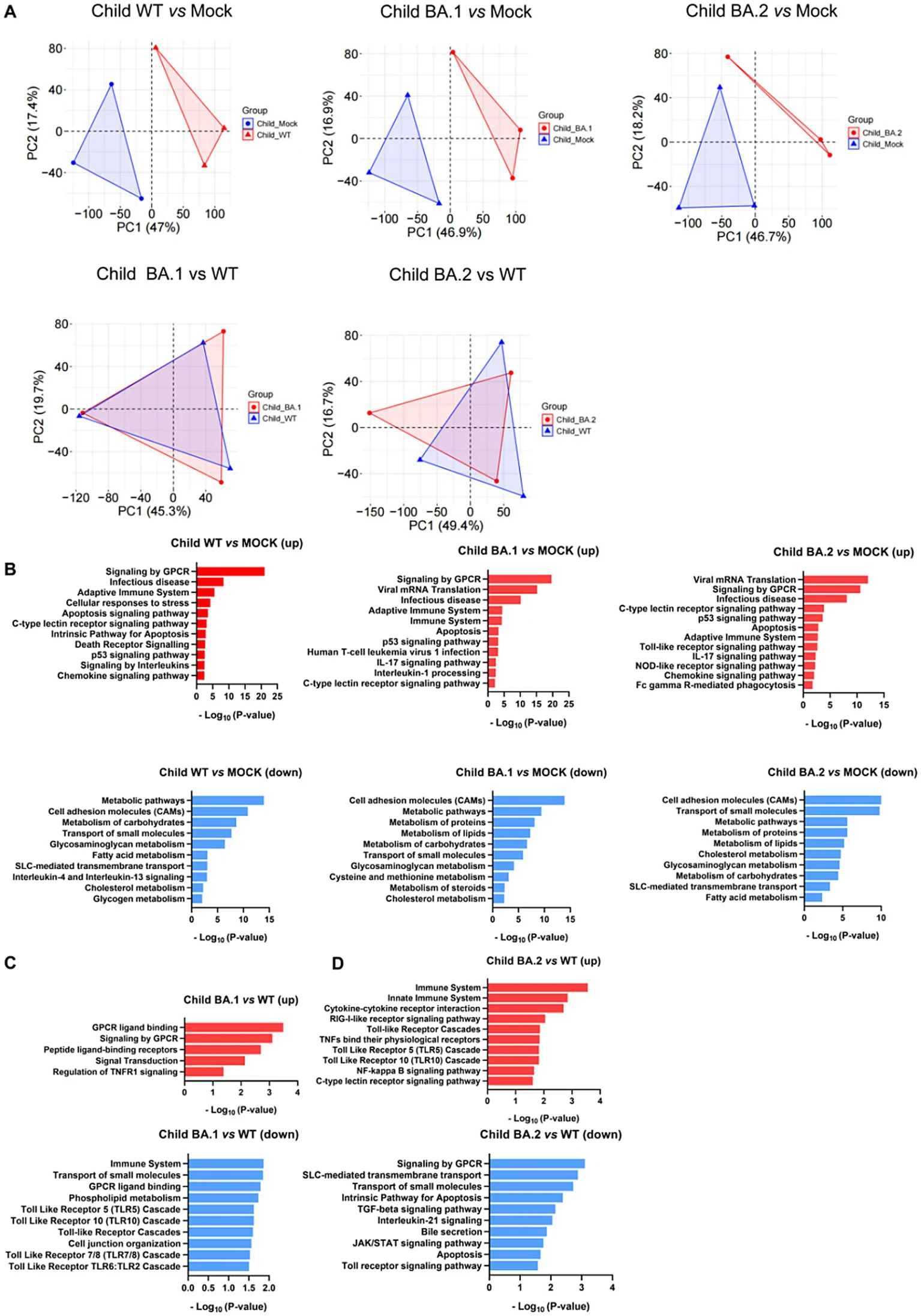
Transcriptional alterations in SARS-CoV-2-infected pediatric liver organoids. **(A)** Upper: PCA of transcriptomic profiles of pediatric organoids infected with WT, BA.1 or BA.2 vs. mock; Lower: PCA of BA.1 or BA.2 vs. WT. **(B)** Pathway enrichment of DEGs in WT/BA.1/BA.2 vs. mock (top upregulated in red, downregulated in blue; ranked by −log10 P). **(C)** DEG enrichment, Child WT vs. BA.1. **(D)** DEG enrichment, Child WT vs. BA.2.

Comparing host responses to Omicron versus WT in children, BA.1 upregulated GPCR ligand binding, signaling by GPCR and peptide ligand-binding receptors relative to WT (Figure 3C, upper), while downregulating the immune system, transport of small molecules, GPCR ligand binding and phospholipid metabolism (Figure 3C, lower). BA.2 upregulated the innate immune system, cytokine–cytokine receptor interaction, RIG-I-like receptor signaling and Toll-like receptor cascades (Figure 3D, upper), and downregulated signaling by GPCR, SLC-mediated transmembrane transport, bile secretion and apoptosis relative to WT (Figure 3D, lower). Altogether, SARS-CoV-2 infection produced a prolonged alteration of innate and adaptive immune gene-expression programs.

### 3.5 Transcriptional analysis of different SARS-CoV-2 variants in adults

We also evaluated infection effects in adult liver organoids. As in pediatric organoids, SARS-CoV-2 infection induced a marked transcriptional shift relative to mock controls (Figure 4A). Pathway analysis revealed significant upregulation of viral mRNA translation, infectious-disease-associated responses, the immune system and IL-17 signaling, with common downregulation of lysosome, protein metabolism and lipid metabolism upon WT and Omicron infection (Figure 4B).

**Figure 4:**
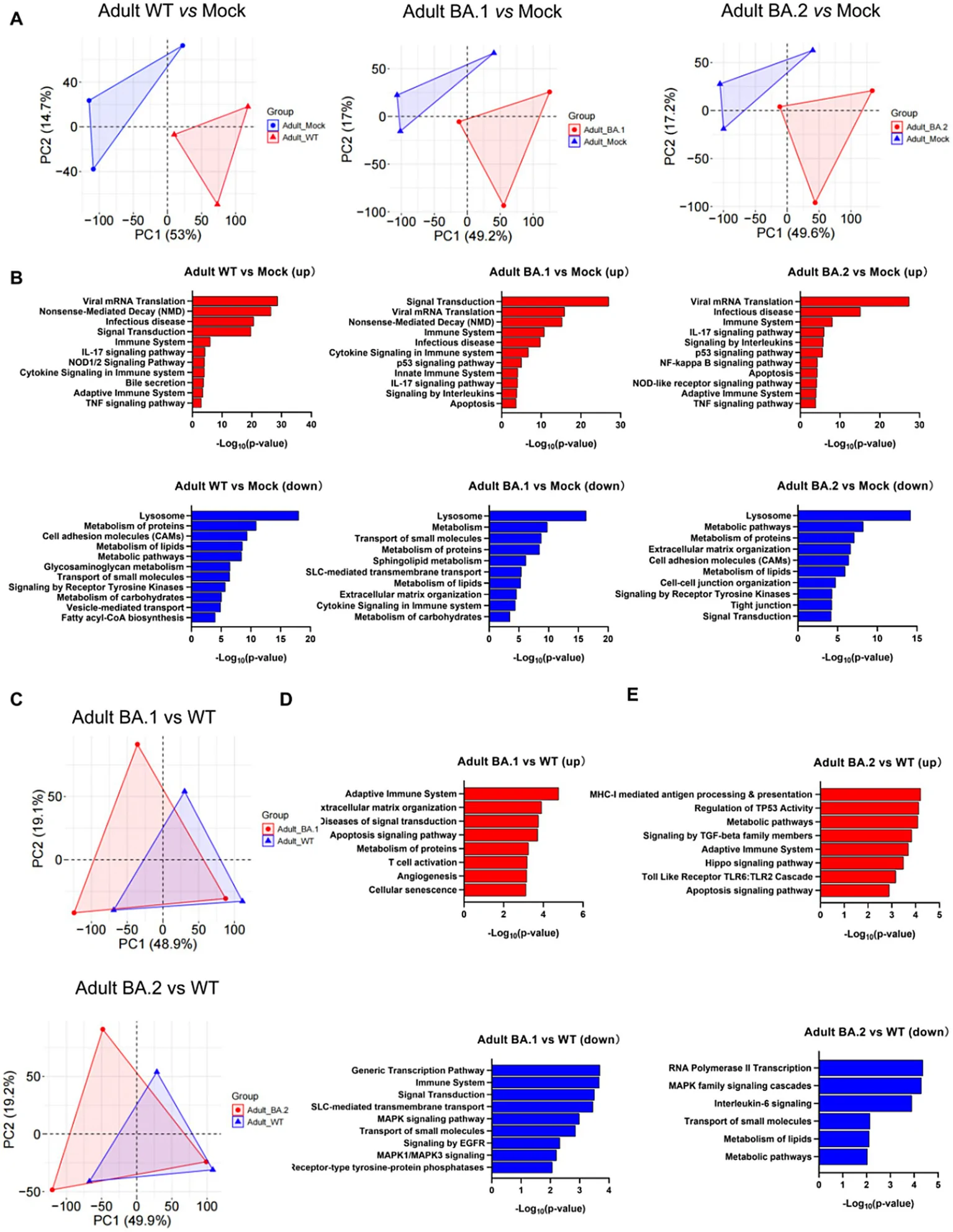
Transcriptomic comparison between SARS-CoV-2 WT and Omicron in adult liver organoids. **(A)** PCA of Adult WT/BA.1/BA.2 vs. Adult Mock (blue = mock, red = infected). **(B)** Pathway enrichment of DEGs vs. mock (red = upregulated, blue = downregulated; x-axis −log10 P). **(C)** PCA of WT vs. BA.1 (top) or BA.2 (bottom). **(D)** Enrichment of genes changed in BA.1 (top) and BA.2 (bottom) vs. WT. **(E)** Enrichment of genes downregulated in BA.1 (top) and BA.2 (bottom) vs. WT.

To further characterize WT–Omicron differences in adults, PCA revealed only modest separation between WT and BA.1 (Figure 4C, upper) and substantial overlap between WT and BA.2 (Figure 4C, lower), suggesting milder transcriptomic divergence from WT in adult organoids. Despite limited global separation, differential-expression analysis identified distinct pathways. Compared with WT, BA.1 upregulated the adaptive immune system, extracellular matrix organization, signal transduction, apoptosis signaling and T-cell activation (Figure 4D, upper), while genes downregulated in BA.1 versus WT were enriched in generic transcriptional programs, MAPK signaling, EGFR pathway activity and transmembrane transport (Figure 4E, upper). In the BA.2 versus WT comparison, WT showed selective upregulation of MHC-I antigen processing and presentation, TP53 regulation, TGF-β family signaling, Hippo signaling and Toll-like receptor cascades (Figure 4D, lower); pathways downregulated in BA.2 included RNA polymerase II transcription, MAPK family signaling cascades, IL-6 signaling and lipid/small-molecule transport metabolism (Figure 4E, lower). Thus, although Omicron BA.1/BA.2 induced transcriptional alterations broadly comparable to WT in adult organoids, it elicited relatively stronger activation of immune and stress-response pathways.

### 3.6 SARS-CoV-2 infection induces a higher innate immune response in pediatric than adult liver organoids

We next explored mechanisms underlying age-dependent differences. SARS-CoV-2 enters target cells mainly through the ACE2 receptor (17, 18). Host-gene analysis revealed a distinct pediatric response characterized by enhanced receptor expression and a pronounced pro-inflammatory signature. Building on the reported interactome identifying ASGR1 as an alternative hepatic receptor (10), heatmap analysis of differentially expressed genes (DEGs) showed that established receptors—ACE2, TMPRSS4 (10) and AGTR2 (19)—were more abundant in pediatric than adult organoids after WT infection (Figure 5A). Concurrently, SARS-CoV-2 robustly induced pro-inflammatory mediators, including the chemokine CCL28, its receptor CCR6 and the cytokine receptor IL17RB (Figure 5A). KEGG analysis identified multiple upregulated immune pathways in infected pediatric organoids, including interferon signaling, cytokine signaling in the immune system, and antiviral mechanisms by IFN-stimulated genes (Figure 5B). The specific upregulation of interferon and broad immune signaling in children suggests that pediatric hepatocytes mount a more vigorous innate immune response than adults.

**Figure 5:**
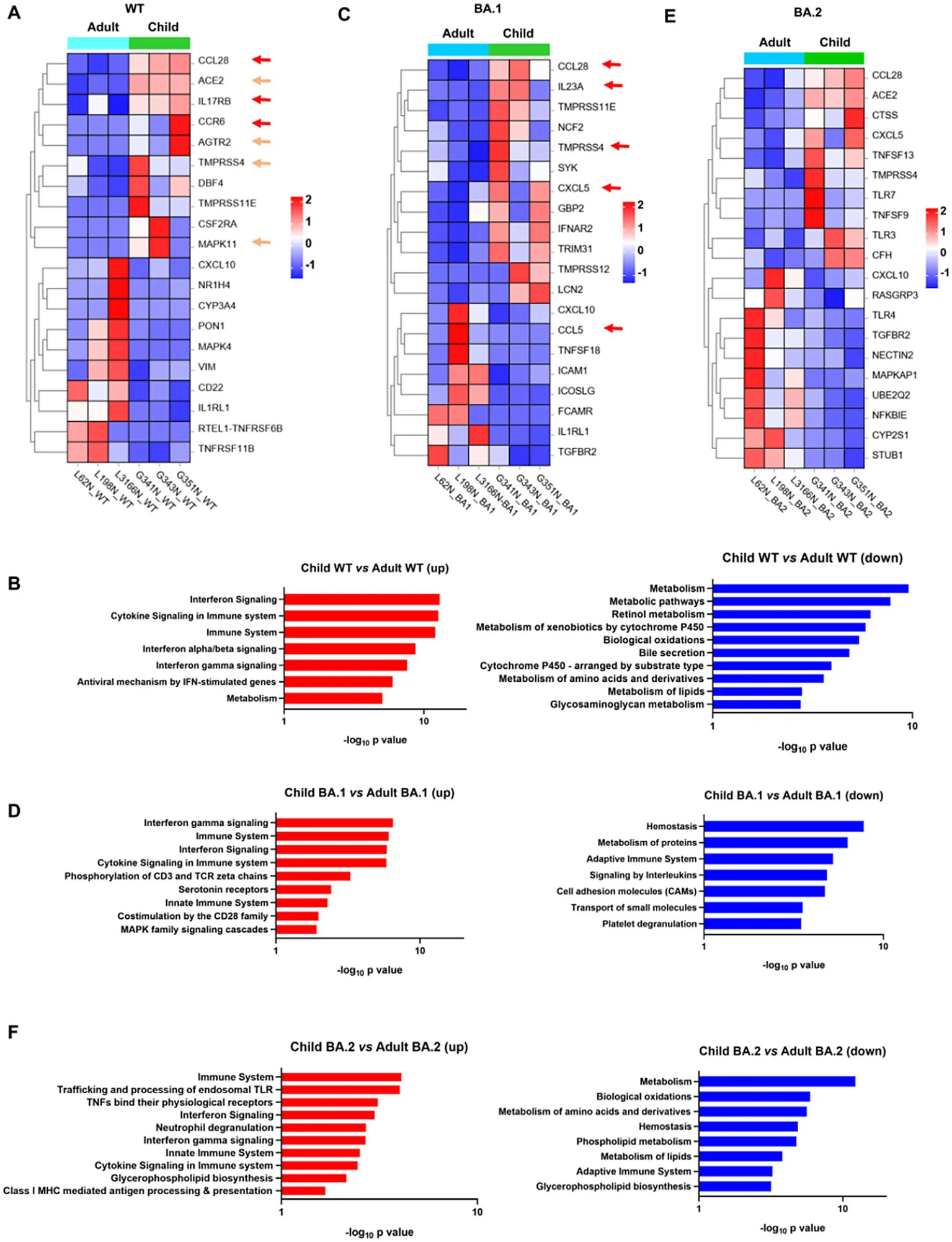
Distinct transcriptional responses to SARS-CoV-2 infection in pediatric versus adult liver organoids. **(A)** Heatmap of gene expression, adult vs. pediatric organoids after WT infection (row-scaled; red = higher, blue = lower). **(B)** Pathway enrichment, Adult WT vs. Child WT (red = upregulated in children, blue = upregulated in adults). **(C)** Heatmap after BA.1 infection. **(D)** Pathway enrichment, Adult BA.1 vs. Child BA.1. **(E)** Heatmap after BA.2 infection. **(F)** Pathway enrichment, Adult BA.2 vs. Child BA.2.

We then examined organoids after Omicron BA.1/BA.2 infection. Cytokine-storm markers such as elevated CCL18 have been reported to correlate with the degree of lung injury by radiomic features (20). Consistent with a dysregulated inflammatory response, BA.1 infection upregulated CCL28 and CXCL5 while downregulating CXCL10 and CCL5 in pediatric versus adult organoids (Figures 5C,E). Expression of the receptor TMPRSS4 remained consistently higher in children across WT, BA.1 and BA.2 (Figures 5A,C,E). KEGG analysis showed that upon Omicron infection, pediatric organoids upregulated interferon-gamma signaling and the innate immune system, whereas the adaptive immune system was downregulated relative to adults (Figures 5D,F). Together, pediatric liver organoids display elevated expression of key entry receptors and more pronounced innate immune activation upon infection, accompanied by relative downregulation of adaptive immune-pathway transcripts. Because our organoids are epithelial/hepatocyte-lineage cultures lacking lymphocytes, these transcriptional changes should not be interpreted as evidence of altered functional adaptive immunity; rather, they underscore age-dependent differences in viral susceptibility and hepatocyte-intrinsic host-response programming.

## 4 Discussion

In this study, we established non-tumorous (tumor-adjacent) liver organoids from pediatric and adult donors to investigate the differential effects of SARS-CoV-2 infection and associated liver injury. The organoids were permissive to both WT and BA.1/BA.2 strains. Although intracellular viral RNA burden was higher with Omicron than WT, no significant difference in viral release into the supernatant was observed between variants. RNA-seq of organoids from three children and three adults, under control conditions and at 48 hpi with WT or Omicron BA.1/BA.2, revealed that Omicron induced a more pronounced immune response in pediatric organoids than WT. Key entry receptors, including ACE2 and TMPRSS4, were upregulated in pediatric organoids, and upon Omicron infection pediatric organoids showed upregulation of interferon-gamma signaling and other innate immune-associated transcriptional programs, with relative downregulation of adaptive immune-associated transcriptional programs compared with adults.

The rise of severe AHUO coinciding with the relaxation of hygiene measures during the pandemic in the UK in 2022 was subsequently linked to increased adenoviral infection, and epidemiological and clinical evaluations suggest an infectious aetiology is most plausible (21). Pathogen screening indicated that more than half of affected children across at least 15 countries tested positive for adenovirus, with UK detection exceeding 70%; serotyping confirmed human adenovirus type 41 (HAdV-41) in 18 cases, detected only in whole blood and not in liver tissue. HAdV-41 is predominantly associated with acute gastroenteritis or respiratory illness and is rarely linked to hepatic failure in immunocompetent individuals. Given the concurrence of SARS-CoV-2 infection and acute hepatitis in children, exploring the connection between current or prior SARS-CoV-2 infection and pediatric hepatitis is essential. Through analysis of pediatric liver samples, we identified an immune-mediated injury pointing to a possible association with prior SARS-CoV-2 infection, suggesting the hepatic disease could manifest as a form of PASC. Organoids are three-dimensional structures that recapitulate key aspects of their organ of origin. Previous studies showed that SARS-CoV-2 productively infects human gut enterocytes (22), and our group previously demonstrated SARS-CoV-2 amplification in lysed organoids and culture supernatant using human hepatobiliary organoids (16). To our knowledge, host-response differences in the liver upon SARS-CoV-2 infection between children and adults, and across strains (WT, BA.1, BA.2), had not been examined; here we used organoids to evaluate these infection effects.

More recently, large case–control genomic investigations have implicated adeno-associated virus 2 (AAV2) as a leading candidate aetiological agent for AHUO. AAV2 DNA and antibody signatures were detected in 81% of AHUO cases compared with 7% of controls and were associated with specific HLA class II alleles, suggesting host genetic susceptibility (23). Independent genomic and proteomic profiling similarly identified AAV2 co-infection with helper viruses (HAdV, HHV-6B), together with hepatic enrichment of T- and B-lymphocytes, HLA class II molecules and complement proteins, supporting a multi-hit model in which abnormal AAV2-derived products trigger immune-mediated liver injury in genetically and immunologically predisposed children (24). Our findings on SARS-CoV-2/Omicron-associated entry-factor expression and innate immune activation should therefore be regarded as a complementary, hypothesis-generating line of evidence rather than an alternative or already-established causal pathway to AAV2-related mechanisms.

We found that the intracellular viral RNA burden of Omicron was much higher than that of WT in organoids from both children and adults. In contrast to our findings in liver organoids, previous studies showed reduced Omicron viral RNA levels in intestinal epithelial cells (25), suggesting tissue-specific differences in variant replication. SARS-CoV-2 tends to affect children differently from adults; most children with acute infection present with mild upper-respiratory symptoms or remain asymptomatic (26). However, as pediatric COVID-19 prevalence increased, a small number developed fever, vomiting, diarrhea, cervical lymphadenopathy and dry lips—features resembling Kawasaki disease and known as multisystem inflammatory syndrome in children (MIS-C), a rare but life-threatening late presentation. In very young children, Omicron reportedly causes less severe disease than Delta; liver damage was associated with SARS-CoV-2 in 77.3% of Delta and 75.0% of Omicron cases, with slight increases in markers of liver function and biliary injury (27).

A broader epidemiological and immunopathogenic perspective on multisystem inflammatory syndrome in children (MIS-C) further supports a host-susceptibility-centered view of pediatric post-infectious complications of SARS-CoV-2. MIS-C incidence has declined markedly across successive SARS-CoV-2 variant periods and with increasing population vaccination coverage, even though its clinical severity per case has remained relatively stable (28). Only a small minority of SARS-CoV-2-infected children ever develop MIS-C, implicating host immune susceptibility, rather than viral factors alone, as a key determinant; this is illustrated by reports of MIS-C occurring in children with inborn errors of immunity, such as Wiskott–Aldrich syndrome (29). The immunopathogenesis of MIS-C is increasingly attributed to persistent viral antigen exposure, with multi-omic studies demonstrating prominent type II interferon and NF-κB transcriptional signatures and elevated circulating Spike protein in MIS-C compared with acute pediatric COVID-19 (30), potentially driven by zonulin-dependent gut-barrier disruption and antigen translocation into the circulation (31). Together with the AAV2/host-genetic-susceptibility evidence discussed above, this literature supports a broader conceptual framework in which host immune and genetic susceptibility — rather than any single pathogen — determines which children develop severe post-infectious inflammatory complications, consistent with the age-dependent host transcriptional differences reported in the present study.

Hepatotropism of SARS-CoV-2, with or without acute hepatitis, is well documented. COVID-19 can elicit significant immune-mediated pathology in many organs, frequently dominated by myeloid–T-cell interactions (32), yet despite the potential for direct hepatocyte infection (33), severe hepatic inflammation is rare. COVID-19-directed immunity elicited after vaccination may also cause liver damage, as suggested by cases of vaccine-induced liver injury in which highly activated CD8 + T-cells, including SARS-CoV-2-specific T-cells, were identified in the liver (34).

Furthermore, our RNA-seq showed that interferon signaling was upregulated in children relative to adults upon Omicron infection. Prior work highlighted an association between clinical severity and the degree of intrahepatic T-cell infiltration, mirrored by strong peripheral-blood immune activation—of potential diagnostic value when liver biopsy is not feasible, and successfully applied via peripheral blood-based immunoprofiling in a North American cohort of pediatric acute liver failure. The highest CD8 infiltration and concomitant peripheral immune activation occurred in the most severe hepatitis, with CD8 + T-cell infiltration linked to interface hepatitis and bridging necrosis (35).

This study has limitations. It is based on a small number of organoid lines and patient samples; the associations are correlative and require prospective epidemiological and mechanistic confirmation, including validation of the age-dependent receptor and immune differences and of the causal role of SARS-CoV-2 in AHUO. In addition, pediatric organoids were derived from hepatoblastoma-adjacent tissue whereas adult organoids were derived from hepatocellular-carcinoma-adjacent tissue, so age-related and underlying-disease-related differences cannot be fully disentangled in the present design. Furthermore, our organoids are epithelial/hepatocyte-lineage cultures that lack lymphocytes and other immune-cell populations, so the innate- and adaptive-immune-pathway transcriptional changes reported here reflect hepatocyte-intrinsic gene-expression programs rather than a functional immune response; likewise, RT-qPCR-based viral RNA quantification does not by itself establish productive infection with infectious virus. In summary, we observed markedly higher Omicron-associated intracellular viral RNA levels and a stronger innate-immune transcriptional response in liver organoids than with WT, and Omicron BA.1/BA.2 induced higher interferon signaling in children than adults. Definitive evidence will be essential to confirm the direct impact of SARS-CoV-2 on children with AHUO, and future research should establish a direct epidemiological link between SARS-CoV-2 and pediatric AHUO.

## Data Availability

The original contributions presented in the study are included in the article/Supplementary material, further inquiries can be directed to the corresponding authors.
