# Supplementary material for: Age-dependent differences in SARS-CoV-2 entry-factor expression and innate immune responses between pediatric and adult liver organoids
Source: Front Public Health. 2026 Oct 23;14:1951670. doi: 10.3389/fpubh.2026.1951670 (PMC13739785; doi:10.3389/fpubh.2026.1951670)
Supplement: Supplementary file 1 [file Data_Sheet_1.pdf]

Supplementary Table S1

Patient ID	Group	Age	Sex	Underlying liver disease	HBV/HCV serology	Fibrosis stage	Steatosis	Prior systemic therapy
341N	Child Liver	6	Male	N/A	N/A	N/A	N/A	Neoadjuvant chemotherapy
343N	Child Liver	7	Male	N/A	HBV	N/A	N/A	Neoadjuvant chemotherapy
351N	Child Liver	13	Female	N/A	N/A	N/A	N/A	Neoadjuvant chemotherapy
62N	Adult Liver	73	Male	Liver cirrhosis,hepatic cyst	HBV	N/A	N/A	N/A
198N	Adult Liver	32	Male	N/A	HBV	N/A	N/A	N/A
3166N	Adult Liver	64	Male	Liver cirrhosis	HBV,HCV	N/A	N/A	N/A
